# Challenges and possible improvements for healthcare teams at outreach clinics in Nepal – a qualitative study

**DOI:** 10.1080/16549716.2024.2385177

**Published:** 2024-08-07

**Authors:** Ingrid Eriksen, Eirin Helene Rasmussen, Biraj Karmacharya, Seema Das, Elisabeth Darj, Maria Lisa Odland

**Affiliations:** a Department of Public Health and Nursing, NTNU, Norwegian University of Science and Technology, Trondheim, Norway; b School of Medical Sciences, Kathmandu University, Dhulikhel, Nepal; c Nell Hodgson Woodruff School of Nursing, Emory University, Atlanta, GA, USA; d Department of Women’s and Children’s Health, Uppsala University, Uppsala, Sweden; e Department of Obstetrics and Gynecology, St Olav’s Hospital, Trondheim University Hospital, Trondheim, Norway; f Malawi Liverpool Wellcome Programme, Blantyre, Malawi; g Institute of Life Course and Medical Sciences, University of Liverpool, Liverpool, UK

**Keywords:** Health providers, Nepal, outreach clinics, perceptions, rural healthcare, qualitative research

## Abstract

**Background:**

All Nepalese citizens have the right to high-quality healthcare services free of charge. To achieve this, healthcare services for the rural population in Nepal need to be improved in terms of personnel, medicines, and medical equipment.

**Objectives:**

To explore challenges and possible improvements healthcare personnel experience when travelling to rural parts of Nepal to provide healthcare.

**Method:**

Data was collected from various health professionals using focus group discussions at Dhulikhel Hospital in Nepal. The data were transcribed and analysed using Systematic text condensation.

**Results:**

Twenty-two professional healthcare personnel participated in five group discussions. Four categories emerged from the collected material: Finding ORC services being underutilised, Wanting to fulfil tasks and do a good job, Facing inadequate resources, and Seeing the need for improved organisation and cooperation. There was consensus that rural clinics are important to maintaining health for the rural population of Nepal. However, there was frustration that the rural population was not benefitting from all available healthcare services due to underutilisation.

**Conclusion:**

Rural healthcare clinics are not utilised appropriately, according to healthcare workers at the rural outreach clinics. Potential ways of overcoming the perceived challenges of underutilising available healthcare services include financial and human resources. The rural population´s health awareness needs to be increased, and the work environment for rural healthcare workers needs to be improved. These issues need to be prioritised by the government and policymakers.

## Paper Context

- **Main findings**: Outreach clinics in Nepal are perceived as underutilised by health providers.
- **Added knowledge**: Increased awareness among rural people on when to seek healthcare, improved work conditions for health providers and collaboration with other health facilities may strengthen the utilisation of offered care.
- **Global health impact for policy and action**: Updated policies reflecting these Nepalese suggestions on strengthening rural healthcare may be useful and benefit other rural populations in similar settings.

## Background

Access to good healthcare services across the globe has been one of the primary goals for both the WHO and the UN for decades [1–3]. The Nepal Interim Constitution of 2007 established that good health is a fundamental right and that all Nepalese citizens have the right to free healthcare services [4]. However, this is not the reality [5]. Therefore, the national authorities have focused on developing a network of rural clinics in local communities. Still, these measures seem insufficient to cover the growing population’s healthcare needs [5,6].

In 2020, Nepal had a population of 29.1 million inhabitants [7]. Poverty is widespread; 25.2% of the population lives below the national poverty line, and the Gross domestic product (GDP) per capita was 1.16 USD in 2020 [7]. This makes Nepal one of the countries with the lowest GDP per capita globally [7]. Nepal’s weak economy makes it challenging for the national authorities to provide infrastructure and quality healthcare services [6,8,9]. Nepal has 8.7 doctors and 34.9 nurses and midwives per 10,000 inhabitants, slightly below the WHO recommendation of 45 doctors, nurses, and midwives per 10,000 inhabitants [3]. The situation is more challenging in rural areas of Nepal, which, despite accounting for almost 80% of the population, has fewer healthcare personnel, healthcare facilities, medical equipment, and available medicines than Nepal’s urban parts [6,8,9]. Despite incentives to get doctors, nurses, and healthcare workers to work in rural areas, the authorities have, to a small extent, managed to get healthcare personnel to take jobs outside the city centres [6]. National authorities have also encouraged the private healthcare sector to contribute to the challenging healthcare situation in rural areas of Nepal [6].

Dhulikhel Hospital, a private, not-for-profit hospital, recognises that central hospitals, like their own, are inaccessible to most of the population in Nepal due to distance, expenses for transport and treatment, and limited bed capacity [6,10]. Therefore, Dhulikhel Hospital has established clinics in various rural areas and currently supports 17 outreach clinics (ORCs) [6,7,10,11]. Healthcare teams of nurses, midwives, doctors, dentists, and public healthcare workers from Dhulikhel Hospital regularly travel to the ORCs to provide medical assistance, and add medical competence.

Since the healthcare services for the rural population in Nepal are scarce [6,8,9], it is essential to consider how various measures aimed at helping this marginalised majority of the population unfold. By understanding the experience of healthcare professionals working in rural areas, it is possible to capture potential issues for improvement. Which may increase the quality of healthcare services offered to a significant proportion of the country’s people living in rural areas. This study aimed to explore challenges that healthcare teams perceive when travelling to rural parts of Nepal to assist with the population's health problems and explore potential areas for improvement.

## Method

### Study design

A qualitative descriptive study design was used, and data was collected through focus group discussions (FGDs). We decided to use FGDs to explore this issue, as they allow participants to interact and give multiple views about the subject and a nuanced picture of the situation they experience [6,12]. The COREQ guideline for qualitative research was followed during the study [13].

### Study setting

This study was carried out in the Community Department at Dhulikhel Hospital in Nepal. Nepal has a federal government system, and health services are under the Ministry of Health and Population [10,14]. The healthcare system in Nepal is organised on several levels, where the ORCs are part of primary healthcare and are structured as health centres [6]. The ORCs are made and driven by Dhulikhel Hospital, which provides services to almost 2.5 million people. Dhulikhel Hospital is situated 30 km northeast of the capital Kathmandu. The hospital introduced the ORCs to care for the rural population, and 17 ORCs were distributed in 10 districts of the country, such as Kavrepalanchowk and Solukhumbhu districts, with a plan to extend the services in other districts [10]. Most of the ORCs are in Kavreplanchowk district, close to Dhulikhel Hospital. The staff working at the ORCs consist of medical officers, nurses, paramedics, and lab personnel. The Community Department gets information from the ORCs when they need a specific specialist, and they conduct regular health camps, like cataract and cervical cancer screening.

### Study participants

A purposive sampling technique was used when recruiting participants from different professions, genders, ages, and work experiences, and assignments were included to ensure various and rich data material contributed to different views of the issues experienced in the ORCs [10,15]. The study’s only inclusion criterion was to recruit participants in healthcare teams from Dhulikhel Hospital, travelling to the hospital’s ORCs to assist the rural population’s health needs. Thirty-five personnel were asked to join the research; 23 accepted, and one did not show up because of an emergency at work. In total, 22 participants were included (Table 1).

**Table 1.** Socio-demographic characteristics of participating health providers.

Group	Age	Gender	Position	Total work experience
1	25–42	5 women	Health assistant, nurse, ophthalmic assistant, auxiliary nurse and midwife (ANM)	7−14 years
2	26–37	2 men, 2 women	Assistant professor, lecturer, nurse	1−11 years
3	24–32	3 men, 2 women	Intern, lecturer, resident, ophthalmic assistant	1−6 years
4	24–39	2 men, 3 women	Resident, consultant, social mobiliser, optometrist	6−16 years
5	38–40	2 men, 1 woman	Associate professor, assistant professor	16−17 years

### Data collection

Data was collected over a period of 2 weeks through five FGDs. According to Malterud, the recommended number of participants is 5–8 [15]. Due to dropouts at short notice because of the participants’ work, there was one group of three participants. Despite this group’s low number of participants, a rich amount of material appeared from the FGDs. Data analysis was conducted concurrently, and when no new topics emerged, data saturation was considered to be reached [16]. The FGDs took place at Dhulikhel Hospital and lasted 55 min on average. The FGDs were moderated by an independent employee at the Dhulikhel Hospitals Research Department with experience in leading FGDs and who used a prepared and pretested interview guide during the discussions. The guide covered topics like a description of their work, challenging aspects of working at the ORCs, and what could be improved. Nepali was used as the communicative language to ensure that opinions and nuances were shared without limitation caused by language problems. The FGDs were audio recorded and translated into English by the local researcher moderating them. The translation was written and controlled by a Nepalese co-author, who checked the recordings from FGDs against the transcriptions to ensure that the transcription quality was approved and that the content was preserved in the English version before the main authors started to analyse the data. When all the translations were finished, the recordings were deleted.

### Data analysis

The collected data was analysed using systematic text condensation, as described by Malterud [15]. The first two authors read the transcripts several times to familiarise themselves with the material and identify preliminary themes related to the prepared interview guide. Next were fragments of the text, meaning units, containing the information on the research questions identified. The meaning units were condensed, coded, and merged into related categories and, lastly, separated into sub-categories. The analysis started during the data collection period in Nepal, and all the co-authors contributed to the discussion of the findings and the analysis (Table 2).

**Table 2.** Example of the analytical process, using systematic text condensation.

Preliminary themes after first readings	Identified and meaning units summarised into codes.		Categories	Sub- categories
	Meaning units	Code		
Despite lack of resources healthcare is provided.	“*Working in ORC, in my opinion, is a form of service. We can at least do something for the patients with the minimum of infrastructure and resources*.”	We can do something with minimum of resources.	Wanting to fulfil tasks and do a good job.	Aim to reach theunreachable.

### Trustworthiness

The credibility of the research was ensured by describing the method in detail, the use of FGDs for collecting the data, the recruiting process, and purposive sampling technique, and the use of systematic text condensation for analyses. The collaboration between the two Norwegian medical students, two international supervisors with experience in qualitative research from various low-income countries, including Nepal, and two local supervisors familiar with the setting secured credibility. The two main researchers’ discussions during data analysis gave several perspectives and a nuanced picture of the material, and the fact that the study was conducted in a short time gave consistency during the data collection period and ensured dependability. The findings can potentially be helpful in other rural areas of Nepal and regions with insufficient resources. Transferability was acquired by describing the local context, background information, and the participants’ characteristics so that readers could evaluate if our findings could be applicable to their contexts.

## Findings

There was a shared agreement between the participants that ORCs are essential healthcare services for the rural population, and simultaneously, a frustration that the service is underutilised. The discussions about challenges and possible improvements led to reflections on increasing the use of the provided healthcare services.

Four key categories emerged from the collected material: Finding ORC services being underutilised, Wanting to fulfil tasks and do a good job, Facing inadequate resources, and Seeing the need for improved organisation and cooperation (Table 3).

**Table 3.** Categories and subcategories developed from the data material after conducting the FGDs.

Categories	Sub-categories
Finding ORC services being underutilised	Meeting insufficient knowledge of health problems Seeing utilisation being affected by economic obstacles
Wanting to fulfil tasks and do a good job	Aiming to reach the unreachable Balancing an unpredictable work situation Fighting against travelling challenges
Facing inadequate resources	Missing essential equipment and medication Encountering shortage of personnel Experiencing inadequate performance of the ORC local staff
Seeing the need for improved organisation and cooperation	Making suggestions for – enhanced information dissemination – relocation of ORC – intensified communication between hospital and ORC levels

### Finding ORC services being underutilised

A common opinion among the participants was the importance of quality healthcare service in rural areas. They perceived that the rural population did not use the available services at the ORCs. They speculated on various possible reasons, such as insufficient health knowledge and physical and economic obstacles, which created difficulties for the rural population in seeking ORCs.

#### Meeting insufficient knowledge of health problems

The participants discussed their views of possible insufficient knowledge of health problems among the rural population, which led to low awareness of when to contact an ORC. This means that the rural population struggles with certain conditions but is unaware of available help or keeps the conditions hidden due to shame. Thus, the rural dwellers do not seek healthcare services in a timely manner. The participants reported treating patients in rural areas was more challenging than in urban areas, and low patient education was perceived as a demanding part of the job at ORCs.

I believe it is difficult to make patients understand due to a lack of education or illiteracy. It takes time to break their mindset, but if you explain to them with a little more time, there are many patients who will understand. (P1, FGD 5)

Therefore, healthcare personnel must have sufficient time with patients to explain the necessity of interventions and the importance of following the treatment plan. The participants wanted to increase their focus on preventive health by raising awareness among the local population. Mobilising healthcare students in awareness campaigns was highlighted as one possible solution.

#### Seeing utilisation being affected by economic obstacles

The participants described several cases where patients with known health problems waited to seek medical help due to economic reasons.

Rural people stay at home even when they are sick. They don’t even have enough money to pay for transport to an ORC or hospital. Because of economical difficulties in rural areas, people will die because they cannot pay for transport and medicine. (P1, FGD 5)

The medical examination is free, but if patients need to buy medicine because of a diagnosed illness, many leave because they cannot afford it. The health providers described that those who are in need to return for follow-up or referrals rarely show up, especially children with severe or chronic conditions.

There is a problem with transportation costs and being away from fieldwork … . these things make it difficult to convince them to come back with their children. These children are easily lost. (P3, FGD 5)

The participants believed this is largely due to unmanageable transportation, food, and accommodation expenses during treatment and the economic challenges of being away from farm work and family. According to the participants, medicines and treatments are reasonably priced at the ORCs but are still too expensive for many. In the past, international non-governmental organisations covered treatment costs for patients, which was suggested as one possible solution to obtain financial support for those who need it the most.

Some patients at ORCs would say they could not afford medicine … Before medicines was donated through international charities, I hope that will happen again. (P2, FGD 1)

### Wanting to fulfil tasks and do a good job

The participants describe positive and negative experiences working on the ORC team. They desire to serve rural patients but encounter difficulties in their work.

#### Aiming to reach the unreachable

Many participants described enjoying working at ORC and being happy they can help patients in rural areas.

Working in ORC, in my opinion, is a form of service. We can at least do something for the patients with the minimum of infrastructure and resources. (P4, FGD 4)

They pointed out that the patients can receive primary healthcare and be referred promptly, preventing their health condition from worsening. They also saw an increased interest from the patients when arranging awareness programs.

The main goal is to reach the unreachable. (P3, FGD 4)

#### Balancing an unpredictable work situation

The participants reported various patients and workloads in ORCs, where they could never predict or prepare for what would meet them. They might leave early in the morning and return in the evening or night without rest. On the other hand, going to the ORCs changes the work environment and is welcome.

There may be nothing to do at times, or there may be an overload and burden that one person cannot handle alone. (P4, FGD 2)

According to the participants, most of the rural population has demanding agricultural tasks. Hence, they can only visit the ORCs after completing their daily agricultural activities. This affects not only those working in agriculture but also the elderly, children, and pregnant women who have no one to escort them to the clinic when their family members are busy with farming. Therefore, the health providers must decide whether to stay longer than planned at the ORC to offer services to patients who arrive late in the afternoon or to return home at the scheduled time. Participants highlighted that outreach activities that last several days are preferable to one-day visits because they can be present at the ORC for at least one afternoon to treat late patients.

It is difficult for us to choose whether to stay and provide services at the ORCs also in the afternoon or return back as planned. Such situations occurs in the most of health camps. A solution is that we can travel one day, examine on next day and return back the day after. (P2, FDG 1)

Another problem described is that there are no fixed times for leaving the meeting point in Dhulikhel. They can be instructed to meet at 6 a.m. but must wait until 9 a.m. because the driver or a team member is late. Those living far from the meeting point have problems getting there early in the morning and face challenges when returning home late. The participants described it as challenging to return to their normal work the day after being at ORCs if they return late in the evenings.

My main issue is that I live in Kathmandu, and after a long day at an ORC I sometimes get dropped off late in the evening at different parking spots along the main road and need to find transport for the last part home by myself. So, travelling to the community is difficult for me. (P1, FDG 5)

#### Fighting against travelling challenges

The roads from Dhulikhel Hospital to ORCs can be challenging. There are landslides, slippery mud, and rising river water levels that cause bridges to collapse. These conditions sometimes make the driver unwilling to continue the trip. Still, at the same time, they are grateful that the drivers prioritise safety. Cars also break down, and sometimes, they must walk the remaining distance to the ORC.

When our vehicle broke down, we had to walk 3 hours to get to the ORC. (P1, FGD 1)

The vehicles are overfilled, and there are often more people than seats and equipment they must bring to the ORCs. A common perception was that travelling is a challenging part of working in the healthcare team. The participants explained that they get exhausted.

We are becoming physically tired if we need to provide services on the same day as travelling. (P3, FGD 1)

The healthcare workers also mentioned that not much food was available at ORCs, and people had been sick from the food served several times. Healthy food and good hygiene would substantially improve the work environment.

### Facing inadequate resources

The participants reported an ongoing lack of resources in the ORC and a gap in knowledge among the staff who work there daily.

#### Missing essential equipment and medication

The participants reported a shortage of equipment, instruments, and medicines.

You can imagine how we would be able to diagnose and treat gynaecological patients in ORCs without speculum examination? Equipment that is required must be available, and only then the patient can utilise services from ORCs. (P1, FDG 2)

ORCs try to provide services, but they do not have necessary resources. There should be a stock of basic medicine. (P3, FDG 3)

The healthcare teams often must bring the necessary equipment and medicines to the ORCs from Dhulikhel Hospital. Several participants reported that there is no system for providing missing equipment and that supplies rarely happen. A common description was that existing equipment and machines in the ORC are often broken and poorly maintained. This means procedures, examinations, and treatments cannot be performed, and patients need to be referred to a higher-level clinic or hospital.

In most of our ORCs, the x-ray machine is not functioning. (P5, FGD 3)

The participants feared patients would lose trust in the ORC, which initially had a good reputation. They shared that due to lacking resources, their treatment is occasionally based on practicality, making them feel like quacks rather than using evidence-based medicine as they are taught. It was suggested that maintenance staff and biotechnicians work on a rotational basis to ensure the inspection and repair of the machines and equipment at ORCs. It was also proposed that a list of the most necessary equipment and medication should always be available in the ORC and supplied when needed.

He [the doctor] told me that he had not conducted a Complete blood count (CBC) for months because of the broken microscope … He said, I am a doctor, but I have only been treating patients empirically. What is the difference between me and quack? (P4, FDG 3)

#### Encountering a shortage of personnel

Participants explained that when the team arrives at an ORC, the work is delegated, and everyone helps. Still, they often must lift the equipment, set up rooms, and prepare the clinical setups themselves before starting the patient consultations. They said that this leaves less time for examinations. There were different perceptions among the participants. Some expressed that human resources in the ORCs are adequate, whereas some think there are signs of the staff being overworked and understaffed.

[ORC staff] … have a lot of work to do as a result of many responsibilities and are not able to follow up patients on time. (P1, FGD2)

#### Experiencing inadequate performance from the local ORC staff

Participants experienced knowledge gaps among the permanent staff at the ORCs, including the medical officers, which could be substandard and inadequate for the delivered healthcare. For example, a nurse was asked about a woman’s contractions in labour and interpreted them as mild when they were moderate to severe.

It means that they either do not have adequate training or are not performing well. (P3, FGD4)

The participants thought that the staff at ORCs should be given refresher training and posted to different departments at Dhulikhel Hospital. This would allow them to update their knowledge.

### Seeing the need for improved organisation and cooperation

During the FGDs, it became clear that there were several areas for improvement by enhancing communication at various levels. The dissemination of information to the local population about available services at ORCs and the cooperation between Dhulikhel Hospital and the permanent staff at ORCs regarding outreach activities. Furthermore, the coordination between ORCs and public healthcare services was seen to improve by strengthening organisational aspects.

#### Making suggestions for …Enhanced information dissemination

When healthcare teams go to the ORCs to organise screening camps or provide specialised treatment, the local community spreads information about the service through posters, radio, and verbal communication. This type of service is often called ‘free camps’ and conveys that the service is free of charge.

Examination is free, but the additional investigation and medication cost money. Such information wouldn’t be broadcast over the radio. If the correct information is disseminated, misconceptions within the rural population will be clear. (P3, FGD4)

Similarly, according to the participants, the services offered daily at the ORCs must be clarified for the local population. The participants believed that patients would have more accurate expectations if more detailed information were shared with the local community about available treatment and expenses. This would further reduce conflict between healthcare personnel and patients.

#### Relocation of ORC

The ORCs were established in their respective locations because there was previously a high population density in these rural areas.

As people migrate to urban areas, there are now fewer people living in the rural areas where we established ORCs years ago. (P1, FGD4)

Many of the ORCs are established on hilltops between villages. This was practical in the past because it meant more people had relatively short walking distances to healthcare services. The impression among the participants is that many patients now prefer to reach an urban hospital since it can be both easier and cheaper to use public transport with the currently improved roads rather than arranging private transport to an ORC. Some participants emphasised that ORCs should be relocated to areas with higher population density. This could increase the patient flow, but as ORCs were originally established to ‘reach the unreachable,’ relocating or reducing the number of ORCs would weaken the service for those who still live in rural areas. The participants emphasised that organising transportation to ORCs with scheduled departures from different villages could compensate for the unfavourable location.

… my suggestion is to have a vehicle to bring the patients from different villages to the ORC for no cost … . people [will] know when to expect transport. It will also make it possible for elderly … . to get help when none in the family are available to escort them. (P1, FDG 4)

Outreach activities are organised according to the ORCs’ reported needs. The participants report many tasks outside patient care when conducting outreach activities. They are primarily responsible for coordinating the outreach activities. They felt this was an unfavourable use of their time and expertise because it reduced their time on patient care.

I think that better communication between ORCs and Dhulikhel Hospital is necessary. Most of the time, there will be a communication gap when performing outreach activities. (P1, FGD 2)

Previously, monthly meetings were held between the coordinators from the Outreach Department at Dhulikhel Hospital and ORCs, and participants suggested that this meeting should be started again. They emphasised that all stakeholders should be included in the meetings, including outgoing healthcare personnel, ORC staff, drivers, and coordinators in the Outreach Department so that all stakeholders regularly will engage in a dialogue to promote solutions to make outreach activities as effective as possible.

#### Intensified communication between hospital and ORC levels

The ORCs were established in the 2000s as a response to the government’s request for help providing healthcare services in rural areas. Today, the government has established Public Health Care Centers (PHCCs) in rural areas. At these facilities, treatment and medication are free of charge if patients have public health insurance. The participants state that resources are insufficient at the PHCCs compared to ORCs.

Patients who come to our ORC are those with diseases that have not been treated by the PHCC. (P5, FGD 4)

The participants explain that if patients need even simple health examinations, such as simple blood tests, X-rays, or medical evaluations by a doctor, they come to an ORC after first visiting a PHCC without these basic services. The participants suggest that the board from Dhulikhel Hospital and PHCCs meet and discuss possible cooperation in each rural area to utilise the total healthcare resources more efficiently to fulfil the local population’s needs. There is also hope among the participants that public health insurance can be used to cover treatment and medication at ORCs, as is the case at Dhulikhel Hospital.

## Discussion

The main finding in this study, according to health personnel at the ORCs in Nepal, is that the available services at the ORCs are underutilised. Several challenges for healthcare workers working at ORCs were identified. Nevertheless, they presented ideas for potential improvements in the work at ORCs.

Efforts are needed to overcome such difficulties, enhancing improved awareness to seek healthcare early for the rural population and, if possible, facilitating transportation. Further, to increase the knowledge level among the permanent ORC staff and to have equipment and medicine in stock and maintained. These changes, high-quality care, and respectful treatment could grow the population’s trust and respect. Increasing the collaboration with other primary health services in the same areas and improving the working conditions for the health workers travelling from Dhulikhel Hospital is seen as essential.

### The outreach clinics are not utilised enough

Low utilisation of health services is complex but can partly be linked to potential distrust in modern healthcare, stigmatised by diseases and avoidance of seeking help, potentially expensive treatment, distance to a clinic and difficulties accessing healthcare facilities [17–19]. The participants emphasised that the location of ORCs is not optimal and may be an obstacle to utilisation. Arranging transportation to ORCs was suggested to assist those unable to reach the clinics. The groups of people with the highest burden of diseases may also have the least capacity to access healthcare [17]. As the health providers in our study mention, assisting these groups in reaching healthcare services may lead to a significant increase in rural health.

The utilisation of available healthcare services also depends on the population’s awareness and attitude towards modern medicine [19]. The participants perceived that rural residents might not be aware of health problems and thus do not contact the ORCs. Two Nepalese studies suggest that illiteracy is the greatest barrier to accessing healthcare services [20,21]. The participants expressed a desire to increase the rural population’s awareness of early warning signs of diseases and the possible outcomes if these diseases are treated in a timely manner.

### Difficulties working at outreach clinics

In all FGDs, the lack of resources was discussed lively. In accordance with other studies, our findings reveal health providers’ perceptions of shortage of equipment, instruments, medicines, and staff in rural areas, which were reported as significant challenges in providing quality healthcare services [6,8,9,22]. The participants were concerned that patients would lose confidence in ORCs as the lack of stability in the services could make the rural population unsure of what help they can expect if they seek healthcare at an ORC. The participants’ concerns are supported by findings pointing out that the rural population experiences limiting factors in rural healthcare due to the lack of diagnostic facilities, unavailability of medicines, and a shortage of human resources [23]. Not knowing if they will receive adequate help reduces the credibility of the healthcare service, which can contribute to a decrease in the population’s willingness to seek professional healthcare. The health providers in our study suggest regularly refilling the most necessary medicines and maintaining machines and equipment to ensure their functionality.

Human resource availability is equally important to medicines and equipment [24]. The participants proposed increasing the knowledge of the permanent staff at ORCs to improve the quality of services. Another study supports that health education interventions improve rural health workers’ performance in diagnosing and treating patients [22]. This will enhance healthcare services quality and increase the staff’s confidence and satisfaction [25]. Continuously, professional development is a crucial factor for the retention of rural healthcare personnel and should be pursued to create stability and quality in healthcare services [23,26].

### The health providers can and wish to provide more care for the Nepali rural population

The desire to be of service to rural patients was reported as an important and challenging factor by the participants. Several participants shared that it was difficult to accept that they could not always provide optimal diagnosis and treatment at ORCs since their decisions often had to be based on empirical evidence. As in other studies, the lack of medical supplies was demotivating for healthcare personnel if they could not utilise their knowledge and provide effective patient treatment according to high-quality standards [26,27]. In line with the participants’ suggestions for possible improvements, there should be a focus on maintaining equipment and regularly refilling basic medicines [22]. This will provide more stable treatment for rural patients while also increasing the confidence of the staff in the quality of their work and their usefulness to the local community.

A challenge in achieving good rural health is motivating healthcare workers, and being recognised for their efforts and knowledge was highlighted as one of the most motivating factors for healthcare workers in a systematic review [27]. It has been shown that simple solutions to improve working conditions increase employee satisfaction and their sense of being valued [25]. Therefore, the participants’ statements about long working days during outreach activities that leave them exhausted, the importance of safe transportation, and providing healthy food should be addressed appropriately. Including healthcare personnel in regular planning and evaluation meetings of outreach activities demonstrates a commitment to valuing their experiences and suggestions for improvement. This will enhance the participants’ sense of value and likely lead to proposals for more efficient use of resources to provide better healthcare services to the rural population.

## Strengths and limitations

The study allows the participants to share challenges from their work when sent to ORCs and allows them to propose improvements to the situation. It is a strength that the study includes all kinds of healthcare professionals. It is a weakness that the study does not include the permanent employees at ORCs, the hospital administration, or the patients using the ORCs. However, this was not the aim of this study. A strength is that FGDs were performed in Nepali and that the moderator is a Nepali citizen familiar with the Nepali health system. Having conducted previous FDGs, probed relevant follow-up questions, and created a safe space for the participants. A limitation might be that the study was conducted by foreign students who could not speak Nepali or were unfamiliar with the local health context. This may have affected the interpretation of the material. Preventive measures to reduce this weakness were participating in the work at ORCs and in Dhulikhel Hospital before the data collection and analysis started to gain better insight and understanding of the context. One of the Nepalese co-authors attended all the FGDs, along with the two main authors, to ensure that there was a shared understanding of the discussions.

## Conclusion

This study aimed to explore the challenges healthcare professionals encounter in their attempt to help the rural population in Nepal. It revealed challenges, perceptions, and suggestions for potential improvements. Their challenges include the underutilisation of available healthcare services and the lack of resources, making it difficult to provide consistent, quality healthcare services. Another challenge to overcome is the distrust of modern medicine among patients. To enhance knowledge about preventive health measures and emphasise the importance of seeking timely healthcare, the government, health policymakers, and healthcare personnel working in rural areas must prioritise financial and human resources.
